# Lane line detection at nighttime on fractional differential and central line point searching with Fragi and Hessian

**DOI:** 10.1038/s41598-022-25032-5

**Published:** 2023-05-12

**Authors:** Limin Li, Weixing Wang, Mengfei Wang, Sheng Feng, Amna Khatoon

**Affiliations:** 1 School of Electrical and Electronic Engineering, Wenzhou University, Wenzhou, 325035 China; 2 School of Information Engineering, Chang’an University, Xi’an, 710064 China; 3 Department Of Computer Science and Engineering, Shaoxing University, Shaoxing, 312000 China; 4 Virtual University of Pakistan, Lahore, Pakistan

**Keywords:** Environmental sciences, Engineering, Mathematics and computing, Optics and photonics

## Abstract

To detect lanes at night, each detecting image is the fusion of the multiple images in a video sequence. The valid lane line detection region is identified on region merging. Then, the image preprocessing algorithm based on the Fragi algorithm and Hessian matrix is applied to enhance lanes; to extract the lane line center feature points, the image segmentation algorithm based on Fractional differential is proposed; and according to the possible lane line positions, the algorithm detects the centerline points in four directions. Subsequently, the candidate points are determined, and the recursive Hough transformation is applied to obtain the possible lane lines. Finally, to obtain the final lane lines, we assume that one lane line should have an angle between 25 and 65 degrees, while the other should have an angle between 115 and 155 degrees, if the detected line is not in the regions, the Hough line detection will be continued by increasing the threshold value until the two lane lines are got. By testing more than 500 images and comparing deep learning methods and image segmentation algorithms, the lane detection accuracy by the new algorithm is up to 70%.

## Introduction

With the continuous development of intelligent transportation system, the safety driving assist system has gradually come into public sight and become popular in human life. In the process of normal driving, when a sudden accident happens, the intelligent assistant driving system provides some services immediately, such as assistant driving decision, emergency braking or emergency warning, to maximize the stability and safety of driving for automation and for drivers, so as to minimize the casualties and economic loss caused by traffic accidents. In an intelligent assistant driving system, a sub-function system, called lane departure warning system (LDWS), has attracted more and more attentions recently^1^.

However, the lane line identification, lane line tracking or lane line departure warning have been the earliest components of image-based driver assistance systems. Since 1990s, those subjects have been researched and implemented for the situations defined by the good viewing conditions and the clear lane markings on road. After then, the robustness for a wide range of scenarios, time efficiency, the accuracy for particular situations and integration into higher-order tasks defines the visual lane detection and tracking as the continuing researches.

LDWS can be basically divided into two categories: vehicle based road classes and infrastructure based ones^2^. Since the former is widely in use and in study, the latter is related to the road construction, and the most roads are un-constructed. The LDWS based on vehicles can be divided into Side view and Forward looking systems^3^. Compared with the Side view system, the Forward looking system has a lot of road information to be applied. It can be utilized normally even on roads without clear lane markings. The key point in the Forward looking system is how to accurately extract lane lines, hence, a huge number of algorithms/methods for lane line detection have been studied by the researchers in the world.

At present, this kind of lane marking detection algorithms based on machine vision and image processing can be divided into two categories: the first kind of algorithms are studied based on the traditional image processing, which is the similar to some kind of linear object detection algorithms^4–6^; and the second algorithms/methods are made based on semantic segmentation^7^. The former mainly utilize the characteristics of the lane line shapes, pixel gradient magnitude and image color features^8^, and they can be classified into similarity based algorithms and discontinuity based ones.

The similarity based algorithms, such as the thresholding algorithms^9^, region similarity based algorithms^10,11^ and others, might be suitable for some special view situations, for instance, some object colors on road can be the cues for lane line extraction^12^; a random finite set at road side can be used in the lane line detection^13^; the morphological distance transform maybe applied to find out lane lines^14^ in a regular mapping road surface; and the fuzzy mathematics is applied into the image segmentation in some kind of vague roads^15^: Ajaykumar et al. made an approach for automated lane line detection by using K-means clustering, the detection effect depends on road quality and weather situations, it is not suitable to complex situations, even the situation is not too complex, the algorithm has to combine other algorithms^16^. For more complicated similarity based algorithm study, Ma et al. researched an algorithm in light on optimized dense disparity map estimation for multiple lane line detection^17^.

In addition to the above similarity based algorithms, a number of algorithms are researched based on discontinuity. In this kind of algorithms, for extracting lane line edge information, it is often to utilize different edge detectors such as Canny, Fractional differential, Gabor, Sobel and Laplacian operators, etc. This kind of algorithms has the fast computing speed and the strong scene adaptability, but it is susceptible to interference from light and obstacles, and they are easy to make the large deviations in the detection results. In general, after edge detection, to make lane line edge sharper, some image enhancement procedures are needed, e.g., Yoo, et al. did the gradient magnitude enhancement for uneven-illumination road traffic images to have the robust lane line detection^18^. After the above image processing, the result image is a binary image, because the lane line edges are discontinuous in a binary image, the lane line maybe a group of line segments, to connect the segments, the standard Hough line transform algorithm is often used, but the results in some cases are not satisfactory. To overcome the shortages, some researchers have studied various straight line and curve fitting algorithms according to the characteristics of lane lines, for instance, Ozgunalp et al. developed such an algorithm for tracing the lane lines based on the vanishing point estimation^19^; Niu et al. made the two-stage feature extraction with a curve fitting function^20^, and in 2021, we studied lane line detection in the raining weather with improved MSR and Hessian matrix for road image enhancement^21^. To well identify lane lines after the above image segmentation, the different versions of Hough transform are published, e.g., Fang, et al. (2018) researched a lane line extraction algorithm on Hough Transform, in their Hough transform, the points conforming to the parallel characteristics, angle and length characteristics, and intercept characteristics of lane line are chosen in a Hough space, where, the selected points are converted into a lane line equation, and the final lane lines are conducted with fusion and property identification^22^; Sun, et al. (2019) made a multiple stage Hough Space computation for lane line detection^23^; and Zhang and Ma (2019) did a lane line detection method by utilizing the Hough transform for the complex environments^24^.

Except for the above image processing based algorithms for lane line detection, recently, the semantic segmentation network based methods are also studied for lane line detection. The semantic segmentation network belongs to Machine learning, and it is a model based on deep convolution network and neural network, its main task is to classify various kinds of pixels in an image, and aggregate the same kind of pixels, then to distinguish different targets in the image^25,26^. For example, the dilated convolution operation was introduced in the U-net model structure, which can greatly increase the local receptive field and gather the multi-scale information without decreasing the number of the dimensions; in the network of deeplab V1, the expansion convolution proportion is further increased, and the conditional random field (CRF) model can be added into the adjacent pixel relationship reasoning; and after that, the deeplab series networks have been developed continuously, in a lot of promoted versions, the backbone network has also been applied, such as the deeplab V3 + network^27^. Nguyen, et al. (2020) proposed a Deep Learning-Gaussian Process method, in the method, a hybrid deep learning-Gaussian process network is suggested to segment a scene image into lane and background regions respectively, and it combines a compact convolutional encoder-decoder net and a powerful nonparametric hierarchical classifier^28^.

Although the good progress has been achieved for lane line detection recently, but there are still some detection problems. One of them is the influence of low quality of lighting, e.g. the lightning at night on road. According to statistical analysis, the road traffic accidents at daytime are less than those at night. The traffic accidents at daytime are mainly caused by a large traffic flow^29^. The occurrence rate of the road traffic accidents at night is 1.5 times of that at daytime. A data shows that 60% of major traffic accidents occur at night^30^. When driving at night, the headlights are often turned on, which can make the drivers completely in the blind spot when the two cars meet, the drivers are unable to see the road conditions in front, which is seriously affecting the driving safety, so it is significant to decrease this kind of road traffic accidents that a vehicle keeps in the two lane lines. Currently, it is a development trend based on LDWS for studying various robust lane detection algorithms which can be adapted to various light conditions and used to overcome the influence of light changes. Borkar, et al.(2009) made the layered approach method for detecting lane lines at night, they firstly extract the regions from a lane line image, then make the image enhancement based on lane shape, subsequently the image of the region is threhsolded, and the lane lines are detected roughly, and finally the lane lines are identified^31^. Song, et al. (2021) studied a deep learning method for the lane detection in low-light situation, in the method, a lane line image is firstly enhanced by a neural network model, then the lane lines are extracted by a deep learning method, and finally the lane lines are identified by applying the KD tree models^32^.

This paper mainly studies a lane line detection algorithm for the night video images. Firstly, an effective lane line detection region is extracted from an image, and then several lane line images in a road traffic video film are merged, and the fused image is smoothed and enhanced by some special developed algorithms. The feature points on the lane line in the enhanced fused image are detected based on Hessian matrix and Fractional differential algorithms in this study. Finally, the lane lines are extracted based on the feature points and image grey level information by a studied recursive Hough line transformation. In order to verify the effectiveness of the new algorithm, it is compared with several traditional algorithms and deep learning methods.

## Lane line image features and multiple image fusion

At night, the contrast of the area of interest will be much lower than that at daytime, within the scope of the street lamp and the place near the lamp, the visual information will be richer, while the information on both sides, especially when it is far away from the lamps, will be weaker.

The features of the lane line image at night are as follows:

1. It is different from the daytime, at night, the lane lines in a road image has weak information. Due to the influence of lights, the white color lane lines are often in gray color. Compared with other parts of the road surface, the color and reflectance of the lane lines are much weaker than that at daytime, that is to say, the difference between the pixel values of the lane lines and that of the road surface on both sides is relatively small.
2. Due to the influence of building shadows, tree shadows, wear of lane marking line itself, strong lights, brake trace lines, fog, haze, sandstorm and rain, etc., the road traffic noise and interference in a lane line image are very strong.
3. In the area close to the vehicle, sometimes, referred to as the nearsighted area, the road information is fuzzy compared with that at daytime, which affects the accuracy of lane line detection; while, in the area far away from the vehicle, due to the strong reflection of street lights, this part of the image reflects the road information well.
4. According to the test of different highway, there are different standards for lane length, width and lane segment spacing, as shown in Table 1. Even if the lane line is not worn, it is difficult to ensure that every video image has the two lane lines, but the probability of acquiring lane lines also depends on the speed of vehicle. When the video is PAL system, 25 images/second can be collected, and when the video is NTSC system, 30 images/second can be acquired. According to Table 2, when the lane line is not worn and the speed of vehicle is 60 km/h, there might be no lane line in 12 images acquired in 30 s on the first class highway, while there might be no lane line in 10 consecutive images on the second class highway. However, if the lane line has been affected by wear or other noises, it may not be possible to see a lane line in dozens of consecutive images, or there is only a small part of lane lines in some images: the line length might be about 10% of the normal length, and the lane line width is only about 1/3 of the original width. No matter what image detection algorithm is applied, it is difficult to detect the lane lines only by using the weak line information. In this case, the best way is to combine the lane line information of multiple images to detect the lane lines.

**Table 1:** Basic dimensions of lane line (*cm*).

Road	Solid line length	Solid line width	Solid line spacing	Dashed line length	Dashed line width	Dashed line spacing
1	600	10–20	900	75	10–15	15
2	400	10–20	600	75	10–15	15
3	300	10–20	400	75	10–15	15
4,5	100–200	10–20	300	75	10–15	15

**Table 2:** Relationship between different vehicle speed and unit time and distance of single image.

Speed: km/h	M/m	M/s	M/image(PAL)	M/image(NTSC)
60	1000	16.7	0.67	0.56
80	1333	22.2	0.89	0.74
100	1667	27.8	1.11	0.93
120	2000	33.3	1.33	1.11

**Figure 1:**
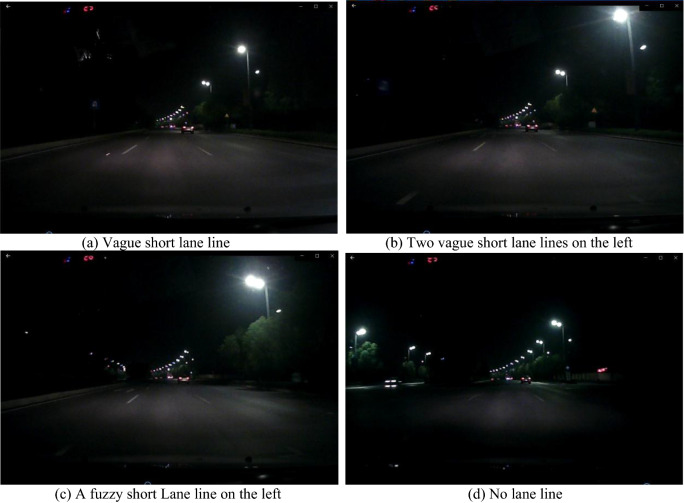
Night traffic images without obvious lane lines.

**Figure 2:**
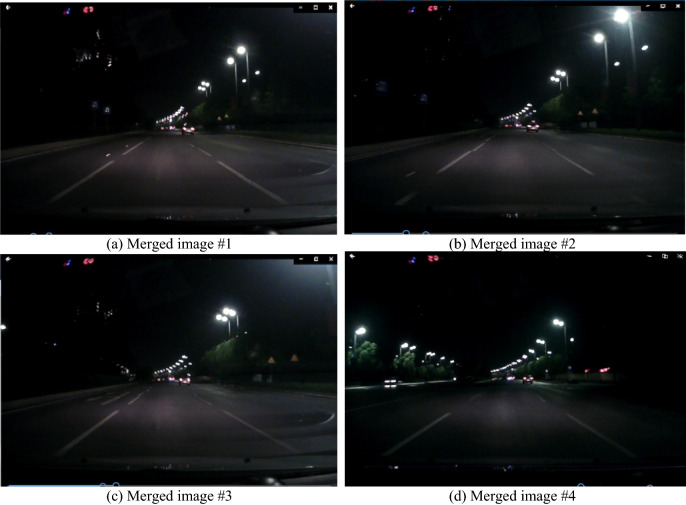
Four obvious lane line images based on the merging results in a video film.

Generally, as the vehicle speed increases, the lane line view will e worse, and the lane line detection will be harder. We take the middle vehicle speed 80 km/h as an example, and the length of a white solid line is 4* m* plus the interval distance of 6* m*, total 10* m*. Eleven images can be obtained continuously by a PAL system to cover the whole 10* m* length in one second, so an image can be selected about every three images and the three images can be used to form the detecting image. During this period, the vehicle moving distance is about 10* m*, which is more than twice of the length of a car, and more than 1/3 of that of a 24 m trailer. During this time interval, the vehicle cannot deviate from the lane line too much and it is difficult to run out of the lane lines too much. In Fig. 1, there are four blurred road images at night, the length of the lane line in each image is very short, or there is no lane line on one side or both sides, which is difficult or even impossible for the lane line detection. The interval of each three of images is within ten images, therefore, after merging the three images, the result in Fig. 2 can be obtained, so the merging result image is better.

## Image smoothing and enhancement based on Fragi and Hessian matrix

**Figure 3:**
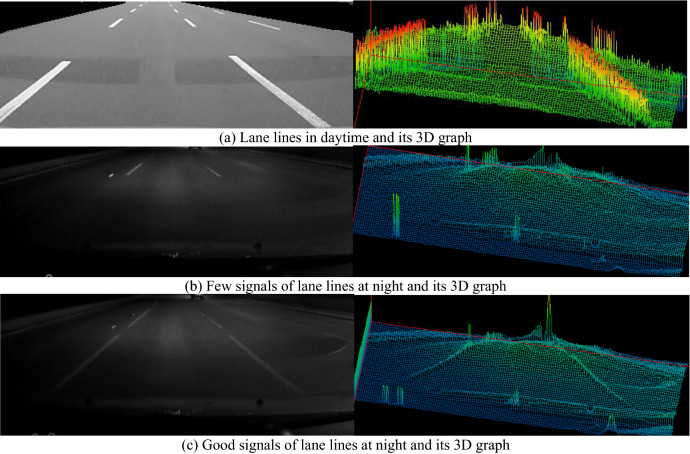
Comparison of lane line information between different situations.

As Fig. 3 shown, the images at night are much different to that at daytime, the images are dark, and the lane line signals are weaker. From the both 2D image and 3D graph, we can see that the lines at daytime are very clear, their gray scale values are much higher than that in background in the 2D image, and the lane line depths at daytime are much deeper than that in the other regions in the 3D graph. In Fig. 3b, when the lane lines are very short, the signals even cannot be seen in the 3D graph. Therefore, we have to do enhancement for the lane lines in the image at night.

Because the contrast of the image at night is poor and the noise is much. Before extracting the lane lines, the image preprocessing should be carried out. The algorithm should includes three basic procedures: image smooth for denoising, contrast stretch and lane line enhancement, to do these, the following Fragi and Hessian matrix based algorithm will be adopted. The specific procedure is as follows^33,34^.

For an array $I:\Omega \to R,\Omega \in R^{2}$, a Gaussian kernel $g(p;\sigma ) = \frac{1}{{\sqrt {2\pi } \sigma }}e^{{ - \frac{{x^{2} + y^{2} }}{{2\sigma^{2} }}}}$ is applied firstly, and then the Hessian matrix corresponding to $I$ at point $p(x,y)$ is set as:

1 $$H_{\sigma } (p) = \left( {\begin{array}{*{20}c} {g_{xx} (p)} & {g_{xy} (p)} \\ {g_{xy} (p)} & {g_{yy} (p)} \\ \end{array} } \right)*I(p)$$

The definition of lane lines is as follows:

2 $$T_{d} (p,\theta ;\sigma ) = t_{d} (p,\theta ;\sigma )*I(p)$$

where, $$t_{d} (p,\theta ;\sigma ) = g_{xx} \cos^{2} \theta + g_{yy} \sin^{2} \theta + g_{xy} \sin 2\theta$$.

The forward filter $t_{f} (p;\sigma ,\psi_{1} )$ and the backward filter $t_{b} (p;\sigma ,\psi_{2} )$ are as:

3 $$t_{f} (p;\sigma ,\psi_{1} ) = t_{d} (x + d\cos (\theta + \psi_{1} ),y + d\sin (\theta + \psi_{1} ))$$

4 $$t_{b} (p;\sigma ,\psi_{2} ) = t_{d} (x - d\cos (\theta + \psi_{2} ),y - d\sin (\theta + \psi_{2} ))$$

where, $\psi_{1} ,\psi_{2}$ are angel adopted for detecting the evidence of lane lines in the neighbor pixels. $d$ is an offset parameter which is set at a appropriate value. It is difficult to contribute enough information when $d$ value is low. It can cause the incorrect segmentation for judging the spurious lane line pixels into the real lane lines.

As the response, the two oriented filters are given by $T_{f} (p;\sigma ,\psi_{1} ) = t_{f} (p;\sigma ,\psi_{1} ) * I(p)$ and $T_{b} (p;\sigma ,\psi_{2} ) = t_{b} (p;\sigma ,\psi_{2} ) * I(p)$ respectively, and the enhanced lane lines are in Eq. (5) :

5 $$T^{*} (p;\sigma ) = T_{d}^{*} (p) + T_{b}^{*} (p) + T_{f}^{*} (p)$$

where, $$T_{d}^{*} (p) = \mathop {\max }\limits_{\theta } T_{d} (p),T_{b}^{*} (p) = \mathop {\max }\limits_{{\psi_{1} }} T_{b} (p),T_{f}^{*} (p) = \mathop {\max }\limits_{{\psi_{2} }} T_{f} (p)$$.

Then we search for the max response at the multi-orientations as the output of image.

**Figure 4:**
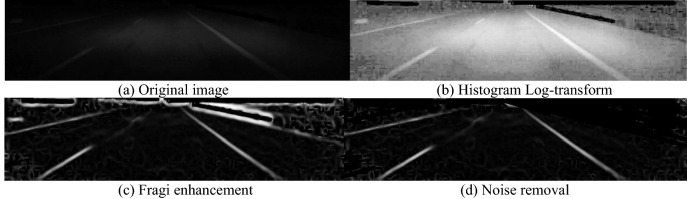
Image smoothing and enhancement for vague lane lines.

In Fig. 4, the typical lane line image enhancement procedure is presented, the original image quality is bad, but their preprocessing results, such as Histogram transformation, Fragi enhancement and noise removal, are satisfactory.

## Search of lane line feature points on fractional differential

Generally, since the gray scales are not uniform^35^ in the lane lines, the more the lane line points we search in a certain area, the lines can be more easily identified. In order to collect as many lane line feature points as possible, we study an algorithm: firstly, the image is inverted to make the lane lines as low gray scales, and then the lane line feature points are detected based on Fractional differential. The specific detection routine is as follows.

**Figure 5:**
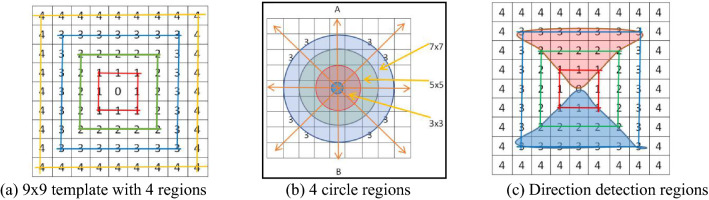
Valley edge detection areas and directions^34^.

In Fig. 5a, it is a 9 × 9 template, in which there are four square areas of different sizes around the center pixel, and the template should be large enough to detect whether the center pixel is a valley edge candidate. For valley edge detection, 81 (9 × 9 template) pixels for calculation may be too large, even if a lot of information is used, but the valley edge detection results are not satisfactory. On the contrary, based on (a), we also test a 7 × 7 square template shown in Fig. 5b, it still needs many pixels (49) for the calculation. Instead of that, we can apply a circular template for the detection, which is more suitable for the actual situation, and can utilize fewer pixels than that in a same sized square area. There are three circular areas (3 × 3, 5 × 5 and 7 × 7) around the central pixel. Since the valley edge point has its four different directions, and the four directions are marked in (b). As an example in Fig. 5c, we mark two trapezoidal areas based on (b), which can be used for valley edge detection in the vertical direction (*AB* in (b)), because we mark "1", "2" and "3" lines in the top trapezoidal area (red color) and the bottom area (blue color ). If the detection pixel "0" is the lowest point, its gray scale should be lower than that in "1" lines. The gray scales in "2" lines should be lower than that in "3" lines. The remaining question is how to calculate the weighted average gray scale value of each line. An example of 5 × 5 templates is given as the follows.

**Figure 6:**
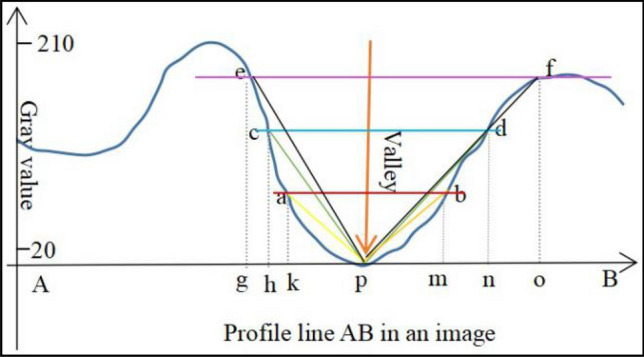
Schematic diagram of lane line detection algorithm.

Suppose there is a lane line center point *P* in the vertical direction in Fig. 5c, we have three detection lines in Fig. 6, they are *ab*, *cd* and *ef* corresponding to lines "1", "2" and "3" respectively in Fig. 6. In the trapezoid area at the top (Fig. 5c), we have orthogonal lines *aP*, *cP* and *eP* in Fig. 6, which meets the conditions of *aP* < *cP* < *eP* in Fig. 6, otherwise *P* is not the center point of the lane line, of course, if the condition *bP* < *dP* < *fP* is not met, it is not enough to determine that *P* is the centerline feature point of the lane line. To determine if point *P* is the centerline feature point of the lane line, we study the following method.

For the gray scale value of each line in Fig. 5c and Fig. 6, it should be a weighted averaging gray scale value, the weight of the central pixel should be larger, and the remaining pixel values should be smaller. Since a lot of literature report that the Fractional differential calculus is good for smoothing thin edges, hence we calculate the coefficients based on Fractional differential. In this study, we use Grümwald-Letnikov (G-L) definition^36,37^ as the following.

For $\forall v \in R$, if a signal $s\left( t \right) \in \left[ {a,t} \right]$, $\left( {a < t,a \in R,t \in R} \right)$, the integral part $\left[ {\text{v}} \right]$ can meet the condition $\left( {m + 1} \right) < m \in Z$, *Z* represents for the continuous derivative of the integer set order; if ${\text{v}} > 0$ and *m* is equal to $\left[ {\text{v}} \right]$, then ${\text{v}}$ order derivative is:

6 $${}_{a}D_{t}^{v} s(t) = \mathop {\lim }\limits_{h \to 0} s_{h}^{v} (t) = \mathop {\mathop {\lim }\limits_{h \to 0} }\limits_{nh \to t - a} h^{ - v} \sum\limits_{r = 0}^{n} {C_{r}^{ - v} } s(t - rh)$$

where, $$C_{r}^{ - v} = ( - v)( - v + 1) \cdots ( - v + r - 1)/r!$$

If the duration $s(t)$ is $t \in [a,t]$, the signal duration $[a,t]$ is divided equally in $h = 1$, the unit equal interval:

7 $$n = \left[ {\frac{t - a}{h}} \right]\mathop = \limits^{h = 1} [t - a]$$

Hence, *v* order fractional order of the differential expression in 1D signal $s(t)$ is deducted as:

8 $$\begin{gathered} \frac{{d^{v} s(t)}}{{dt^{v} }} \approx s(t) + ( - v)s(t - 1) + \frac{( - v)( - v + 1)}{2}s(t - 2) + \frac{( - v)( - v + 1)( - v + 2)}{6}s(t - 3) + \cdots , + \frac{\Gamma ( - v + 1)}{{n!\Gamma ( - v + n + 1)}}s(t - n) \hfill \\ = a_{0} s(t) + a_{1} s(t - 1) + a_{2} s(t - 2) + a_{3} s(t - 3) + \cdots , + a_{n} s(t - n) \hfill \\ \end{gathered}$$

The *n* + *1* non-zero coefficient values can be in order as:

9 $$\left\{ {\begin{array}{*{20}l} {a_{0} = 1} \hfill \\ {a_{1} = - v} \hfill \\ {a_{2} = {{( - v)( - v + 1)} \mathord{\left/ {\vphantom {{( - v)( - v + 1)} 2}} \right. \kern-\nulldelimiterspace} 2} = (v^{2} - v)/2} \hfill \\ {a_{3} = ( - v)( - v + 1)( - v + 2)/6 = ( - v^{3} + 3v^{2} - 2v)/6} \hfill \\ \begin{gathered} a_{4} = ( - v)( - v + 1)( - v + 2)( - v + 3)/24 = (v^{4} - 6v^{3} + 11v^{2} - 6v)/24 \hfill \\ ...... \hfill \\ \end{gathered} \hfill \\ {a_{n} = \Gamma ( - v + 1)/n!\Gamma ( - v + n + 1)} \hfill \\ \end{array} } \right.$$

We make the absolute values: $a_{0} = 1$,$a_{1} = \left| { - v} \right|$,$a_{2} = \left| {\left( {v^{2} - v} \right)/2} \right|$, when ${\text{v}} = {0}{\text{.5}}$, we obtain $a_{1} = 0.5$, $a_{2} = 0.{1}25$, in order to remove the decimals, for line “1”, we enlarge all digits for 2 times, then we got $b_{0} = {\text{2a}}_{{0}} = 2$,$b_{1} = {\text{2v}} = {1}$; and for line “2”, we enlarge all digits for 8 times, then we obtain $c_{0} = {\text{8a}}_{{0}} = {8}$,$c_{1} = {\text{8v}} = {4}$,$c_{2} = {\text{8u}} = 1$.

The valley or ridge detection algorithms have been used in different applications, but for this application, we study a special algorithm, which is different to others^38,39^, as described as the follows.

**Figure 7:**
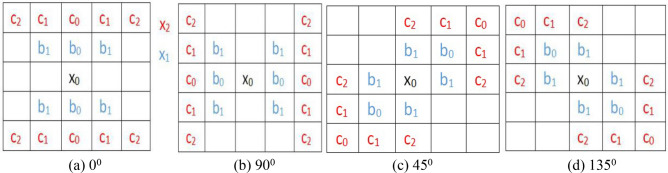
Template for line marking in four directions.

As Fig. 7 shown, the templates for four directions are illustrated. Where, we define detecting point or central pixel as $x_{0}$, line “1” as $x_{1}$ and line “2” as $x_{2}$, in the vertical direction (Fig. 7b), the top part (Fig. 5c, or red color trapezoid region) is taken as an example for the valley edge point detection. $f\left( {i,j} \right)$ is a gray scale image for input, and $g\left( {i,j} \right)$ is the binary image for output.

$$x_{0} = f\left( {i,j} \right)$$

10 $$x_{{1}} = \left[ {b_{0} f\left( {i{ - 1},j} \right) + b_{1} (f\left( {i{ - 1},j - 1} \right) + f\left( {i{ - 1},j + 1} \right))} \right]/\left( {b_{0} + 2b_{1} } \right)$$

11 $$x_{{2}} = \left[ {c_{{0}} f\left( {i{ - 1},j} \right) + c_{1} \left( {f\left( {i{ - 2},j - 1} \right) + f\left( {i{ - 2},j + 1} \right)} \right) + {\text{c}}_{{2}} \left( {f\left( {i{ - 2},j{ - 2}} \right) + f\left( {i{ - 2},j + {2}} \right)} \right)} \right]/\left( {c_{0} + 2c_{1} + {\text{2c}}_{{2}} } \right)$$

12 $$y = \left( {x_{2} + x_{1} } \right)/2 - x_{0}$$

In the vertical direction, we have two values (at the top region and the bottom region, see Fig. 5c), we call them as $y_{{ + {9}0}}$ and $y_{{{ - 9}0}}$, if $y_{{ + {90}}} > 0$ and $y_{{ - 90}} > 0$, we have $y_{{{90}}} = y_{{ + {90}}} + y_{{ - 90}}$. In the same way, we calculate the other three directional *y* values. Then, we compute:

13 $$z = \max \left( {y_{0} ,y_{45} ,y_{90} ,y_{135} } \right)$$

To output a gradient magnitude image, we do:

14 $${\text{If}}\,z > 0,\,g\left( {i,j} \right) = z,\,{\text{otherwise}}\,g\left( {i,j} \right) = {0}$$

If we output a binary image directly, when we set a threshold *T*, we can do:

15 $${\text{If}}\,z > T,\,g\left( {i,j} \right) = 255,\,{\text{otherwise}}\,g\left( {i,j} \right) = {0}$$

**Figure 8:**
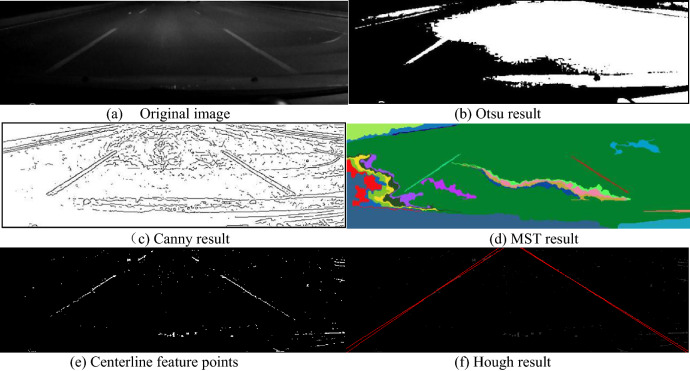
Lane line detection with different algorithms.

It is normal that an original image include a lot of noise which will affect valley edge detection result. One simple way for reducing the noise is to use a smoothing filter such as the Gaussian smoothing function, which has a width parameter sigma, often referred to as the scale space parameter. The choice of sigma depends on white spot size distribution. Figure 8 gives the comparison between the new algorithm and other traditional algorithms for the two lane lines in the image at night.

In Fig. 8a, there are two vague lane lines in the original image (the vehicle speed is 60–80 km/h). Otsu thresholding segmentation result shows that the image illumination is uneven, the gray scale value of the bottom left corner is lower, and the gray scale value of the middle part is shallow, as shown in Fig. 8b. In Fig. 8c, the image is a binary image obtained by the Canny edge detector. For the Canny operation, we give a lower threshold value, and the double edges of most areas of lane lines are displayed^40^. However, there is too much noise in the result image, which is difficult to remove by the post-processing functions. In Fig. 8d, the Minimum Spanning Tree (MST) algorithm (Graph based algorithm)^41^ is applied to segment the image. In the result image, some segments of the two lane lines are detected (the green segment on the left and the purple segment on the right), but the noisy segments are thicker and larger, and some post-processing functions may be needed. In Fig. 8e, the new algorithm detects most of the feature points of the lane centerlines, and it is easy to detect two lane lines (red line) with the Hough transform algorithm.

## Experiments and analysis

**Figure 9:**
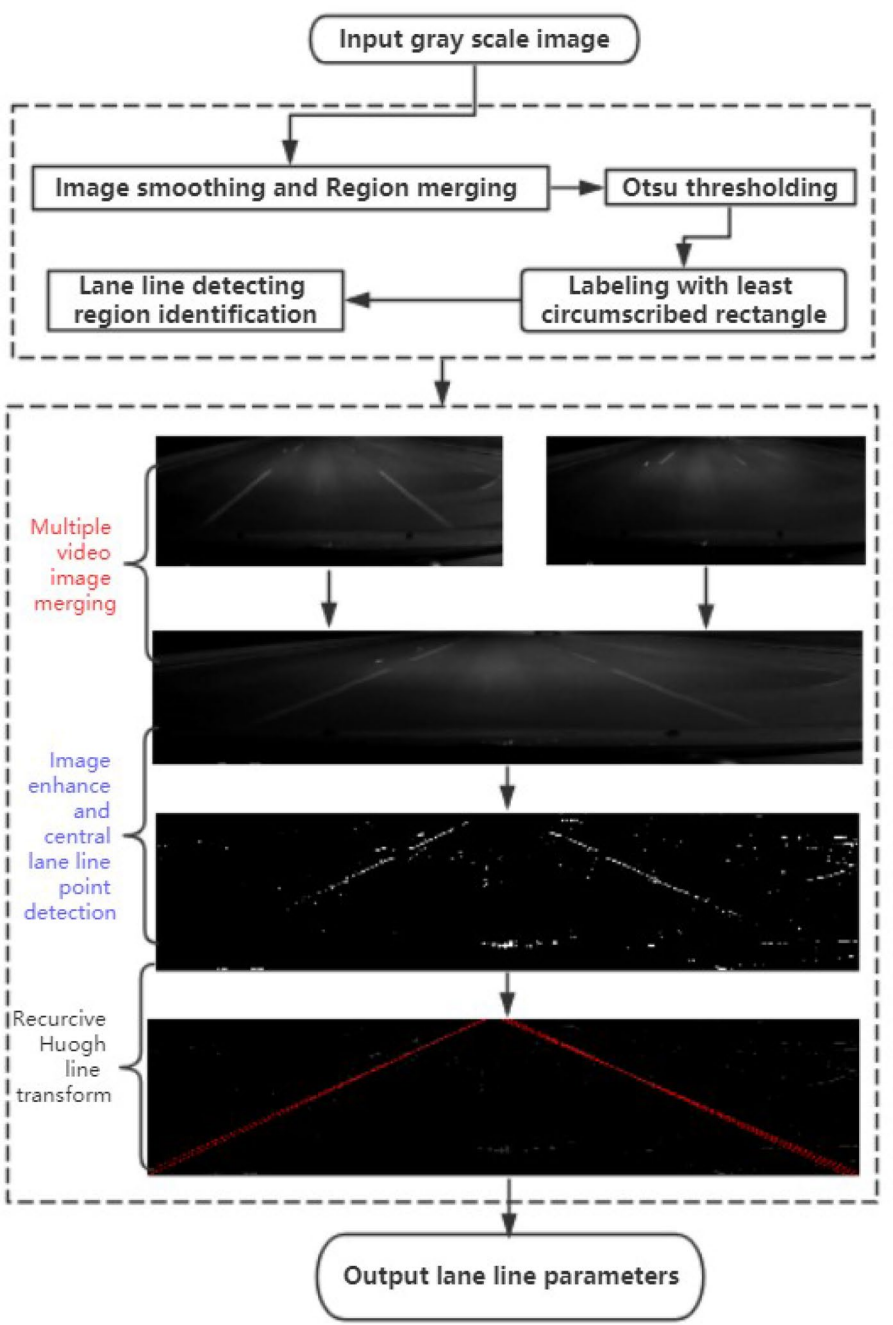
Workflow of lane line detection.

As above description, the lane line detection at night is harder, hence we study a new method for the lane line extraction, and the method working procedure is presented in Fig. 9. The method mainly includes two parts as the dashed line rectangles shown, the first part is to extract valuable detecting regions, which can reduce the method calculation burden and remove noise which can greatly affect the detection results.

In Fig. 9, the 40 min video film from highway at night is taken as examples (vehicle speed is 80–100 km/h). The hundreds of road images are tested by the new method which includes several algorithms. The typical examples and the algorithm comparison results are presented in the following sections.

### Comparing new algorithm to traditional image processing algorithms

**Figure 10:**
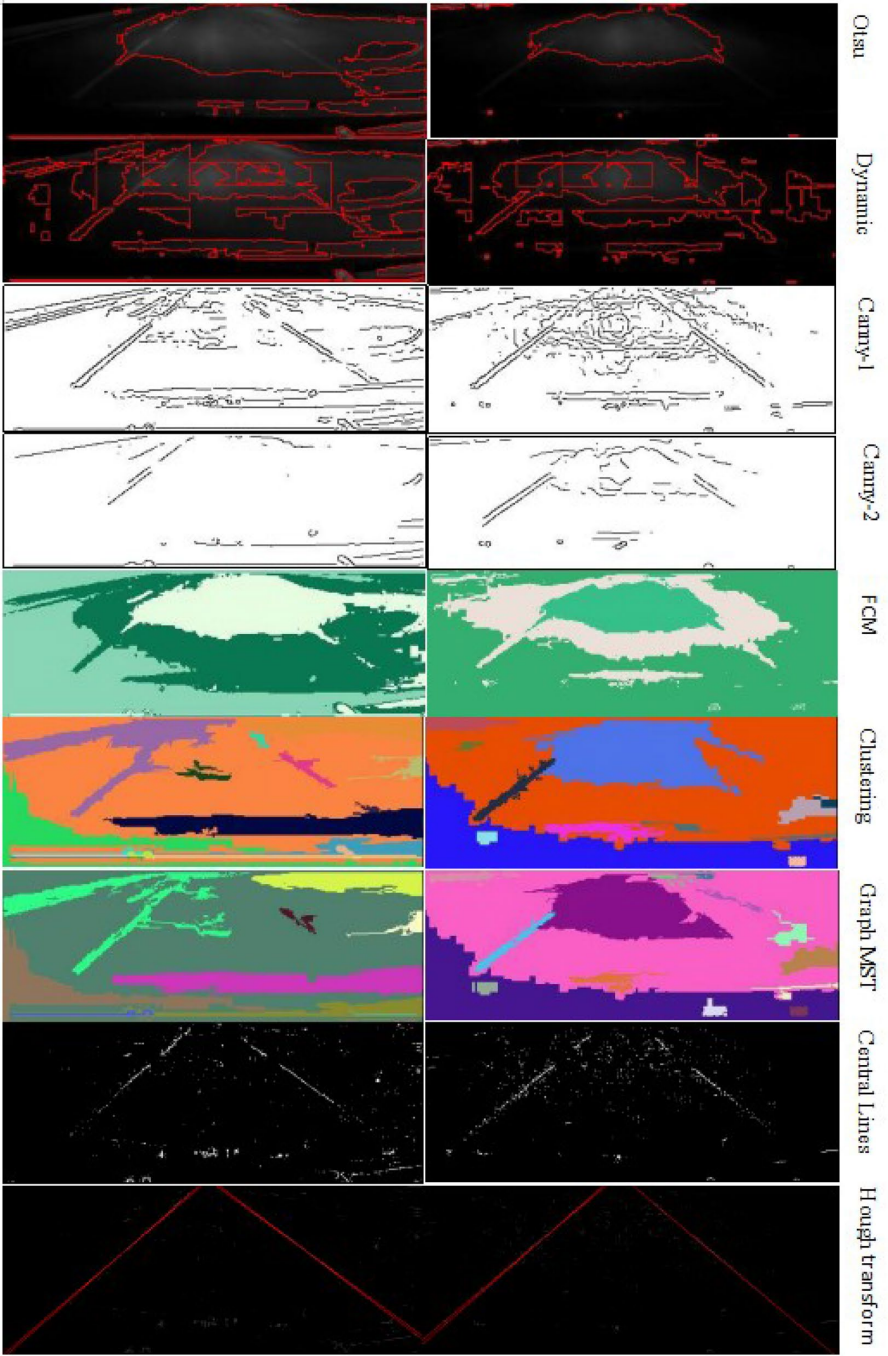
Comparison of several lane line detection algorithms for two images.

In Fig. 10, there are two typical pre-treated night traffic road images. Most of the noises in the image have been smoothed out and the lane lines have been enhanced. However, the obvious features are that the image is darker, and the contrast is poor, and the lane lines are vague. The gray scale histograms of the two images show that the gray scales of most pixels in the images are less than 64. In this case, it is almost impossible to detect lane lines only based on image histograms. Therefore we try some other basic and conventional traditional algorithms to deal with these two images.

There are eight kinds of algorithm processing results. The basic segmentation algorithms based on similarity are global thresholding and dynamic thresholding. The global thresholding algorithm we use here is Otsu, while the dynamic thresholding algorithm divides the image into 9 × 6 sub-windows for Otsu thresholding in each sub-window. From the first row of Fig. 10, it can be seen that the global thresholding algorithm can only extract the brighter area in the upper and middle parts, some lane line segments are included in the parts, while the other parts are included in background, no complete lane line is detected, so this method is invalid. In the second row of Fig. 10, there are a lot of targets (red line outline) in each image, and only some lane line segments are detected. It is difficult to separate these lane line segments from other regions because there are many targets adhered around, and even if these segments are separated from the other regions, it is difficult to form a complete lane line or even half a lane line. Therefore, the algorithm is not suitable for this type of images.

The third and fourth rows in Fig. 10 are Canny detection results with different high and low thresholds^40^. It can be seen that the image segmentation algorithms based on discontinuity are superior to the algorithms based on similarity. When Canny's thresholds are low, although the edges of the targets can be detected, but there are many noise edges in the images, that is over segmentation or over detection, which is hard or even impossible for the post-processing. However, when the thresholds are selected as higher values, although the noise edges are much less, the boundaries of some targets are missed, that is under segmentation or missing detection. So, it is not enough to provide complete information for lane line identification.

The fifth row is for the results of FCM (fuzzy clustering)^42^. It has the similar effect with the global thresholding results, the regions with high gray scales in the upper part and the middle part are detected as targets, and the lane line (or part of the segment) is not fully extracted. Although the algorithm is effective for complex multi-target image segmentation, it cannot achieve the desired effect for the situation of slender target and low contrast images. The sixth row is for the detection result images of clustering analysis^42^. Different from FCM, it can segment multi-targets as many as possible, so it can extract some lane lines (or some line segments), but the lane line extraction is not completed, or lane lines are fused into other targets, only some points (spots) and a part of lines (line segments) are detected. Hence, although its detection results are better than that by the above-mentioned FCM algorithm, but for this kind of special images, we should have some improvements for the algorithm to get better results.

In the seventh row, the results are obtained by MST (Minimum Spanning Tree) algorithm^41^. Compared with the above Clustering analysis algorithm, the effect of image segmentation is improved, but the extraction of lane lines is not completed. Even if the Hough transform is used to detect lane lines in the post-processing, it is difficult to achieve the required effect due to the impact of noise targets, so there is still a lot of room for improvement.

The eighth row presents the results based on the detection of the characteristic points on the lane line by the algorithm studied in this paper. Its main idea is to detect as many feature points as possible on the lane lines, to find as many points on the canyon line or the center of the potholes in the canyon as possible.

For the algorithm comparison, three parameters are listed in Table 3, and the best result image should have clear lane lines with less under-detection and over-detection.

**Table 3:** Eight algorithm detection results in Fig. 10.

Algorithm	Otsu	Dynamic	Canny-1	Canny-2	FCM	Clustering	MST	New
Lane lines	0%	15%	40%	25%	10%	40%	20%	95%
Under-detection	100%	75%	30%	30%	90%	25%	20	0%
Over-detection	100%	50%	40%	10%	70%	15%	25%	0%

In Table 3, “Lane lines” means that percentage of lane lines is clearly detected, the higher it is, the better the result; “Under-detection” means that the percentage of lane lines is not detected, the greater it is, the more lane lines are not detected; and “Over-detection” means that the percentage of lane lines is cut into different objects, the grater it is, the more objects are on the lane lines.

Although the points found are not necessarily continuous, they are mostly concentrated on or near to the lane lines. For the new algorithm, compared with Canny or other differential operators, the algorithm does not generate too much noise and false edges, which lays a good foundation for the subsequent recursive Hough transform. The ninth row is for the corresponding recursive Hough line detection results^23,24^. The detection results can fully meet the requirements of lane line detection. Hence, we detect hundreds of the nighttime lane line images based on the detection of feature points on the lane centerline and the recursive Hough line transformation. Because of the difference of images, for the Hough transform, in some images, only one lane line is extracted first, and when Hough transform is continued more than once, two lane lines are detected (finally, the innermost line is taken as lane line), which is called the recursive Hough transform. It can be seen from Fig. 10 (last row) that in some lane lines there are multiple red lines. In addition, the bottom or upper parts of the images may be detected as a near horizontal red line due to the influence of noise (sixth row), but it can be judged that it is not a lane line according to the angle of the line segments.

### Comparing new algorithm to deep learning method

Li 2020^27^ studied a new semantic method (a Deep learning method), called Deeplab V3 + network, which was compared with other similar three semantic methods as Table 4. The image data is from Tusimple (https://github.com/TuSimple/tusimple-benchmark/issues/3), where, the training set includes 3626 images, and testing set involves 2782 images.

**Table 4:** Comparison of test results of different models^27^.

Data set type	Method	Recall rate	Accuracy
Straight set	ENet	75.5%	73.4%
	SCNN	95.2%	93.2%
	Deeplab v3+	95.4%	93.4%
	Deeplab V3 + network	95.8%	94.8%
	ENet	71.2%	70.5%
Corner set	SCNN	94%	93.5%
	Deeplab v3+	94.2%	93%
	Deeplab V3 + network	95.5%	94.5%
Worn lane set	ENet	70.2%	69.5%
	SCNN	93.8%	91.5%
	Deeplab v3+	92.8%	90.6%
	Deeplab V3 + network	93.5%	92.8%
Night environment	ENet	40.5%	38%
	SCNN	70.5%	68.1%
	Deeplab v3+	71.2%	65%
	Deeplab V3 + network	65.5%	62.4%

For the Accuracy and Recall in the Table 4, the definitions are as the follows.

Accuracy: It is the proportion of the sample in the test set that can be accurately detected, and it can be expressed as the follows.

16 $$Accuracy = {{(TP + TN)} \mathord{\left/ {\vphantom {{(TP + TN)} {\left( {TP + TN + FP + FN} \right)}}} \right. \kern-\nulldelimiterspace} {\left( {TP + TN + FP + FN} \right)}}$$

Recall: It is the proportion of all positive samples in the test set that are correctly identified as positive samples, and the parameter can be presented as:

17 $${\text{Re}} call = {{\left( {TP} \right)} \mathord{\left/ {\vphantom {{\left( {TP} \right)} {\left( {TP + FN} \right)}}} \right. \kern-\nulldelimiterspace} {\left( {TP + FN} \right)}}$$

where, *TP* (True positives): the positive samples are correctly identified as positive samples, and the marked lane lines are accurately identified; *TN* (True negatives): the negative sample is correctly identified as a negative sample, the marked lane is not identified, but he system mistakenly thinks it is a lane line; *FP* (False positives): False positive samples, i.e. negative samples are incorrectly identified as positive samples, non Lane images are identified as lanes, or multi Lane images are not fully identified; *FN* (False negatives): False negative samples, i.e. positive samples are erroneously identified as negative samples, and data without lanes are erroneously considered as lane data.

It can be found from Table 4 that, the results are greatly affected by the night environment lighting, resulting in the decline of detection accuracy, the analysis result shows that due to the comprehensive influence of various light sources such as neon lights and street lights in the urban night environment, all the methods caused certain misjudgments to the lane line area, resulting in a lower overall detection success rate.

**Figure 11:**
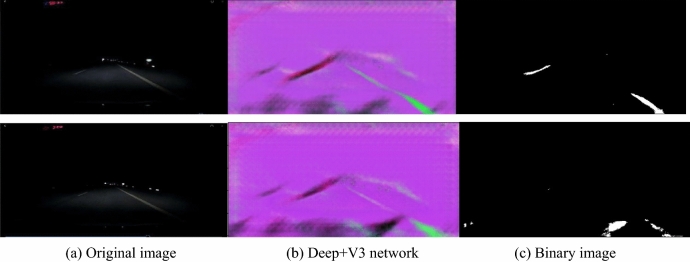
Night lane line detection by Deep + V3 method^27^.

In order to make the validation for the algorithm in this study, the new algorithm is compared to Li’s method—Deep + V3 network. The two typical nighttime lane line images are selected as shown in Fig. 11.

**Figure 12:**
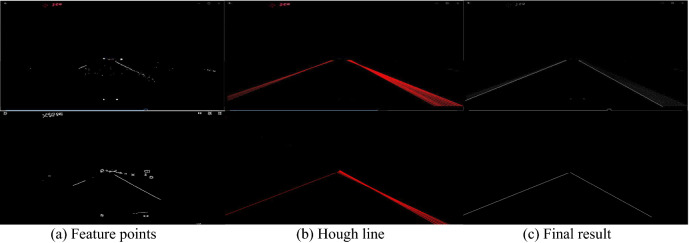
Extraction lane line in images in Fig. 11 by new algorithm.

In Fig. 12, the original images are from Fig. 11. It shows three step image processing procedure: lane line center line points, the recursive Hough transform and final results. Comparing to the results in Fig. 11, the new algorithm is much better than Deep + V3 network method.

At present, the data sets about lane line detection include CuLane data set, KITTI data set, TuSimple data set and Baidu Apollo lane line pixel data set^27^. KITTI data set contains less lane line feature data, the lane structure is not obvious, and the road environment irrelevant information is too much to meet the requirements of this paper. Baidu Apollo data is mainly aimed at the driving environment of automatic driving, and there is no relevant annotation requirement for the lane line structure. Therefore, in this paper, we select TuSimple data set CuLane data set as the main test and training objects.

To evaluate the new algorithm detection results, we took m0re than 400 lane line images, 200 images from TuSimple data set^43^ and 200 images from CuLane data set^44^, and the algorithm mainly is compared to a semantic method (Deeplab V3 + network)^27^, and the testing and comparison results are presented in Table 5, where, in the daylight, the results between two methods are almost the same, and in the night, the new algorithm has increased the Accuracy and Recall much than the semantic method.

**Table 5:** Straight lane line detection accuracy and recall in daylight and night.

Method	Evaluation	Daylight	Night
Deeplab V3 + network^27^	Accuracy Recall	98.1% 99.3%	51.3% 53.8%
Algorithm in this study	Accuracy Recall	98.2% 99.01%	53.1% 56.5%

**Figure 13:**
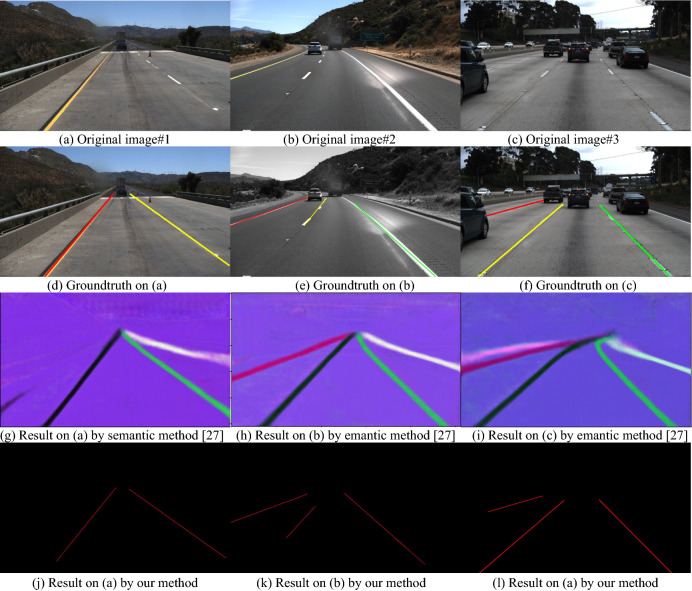
Detection results based on TuSimple data set^43^.

**Figure 14:**
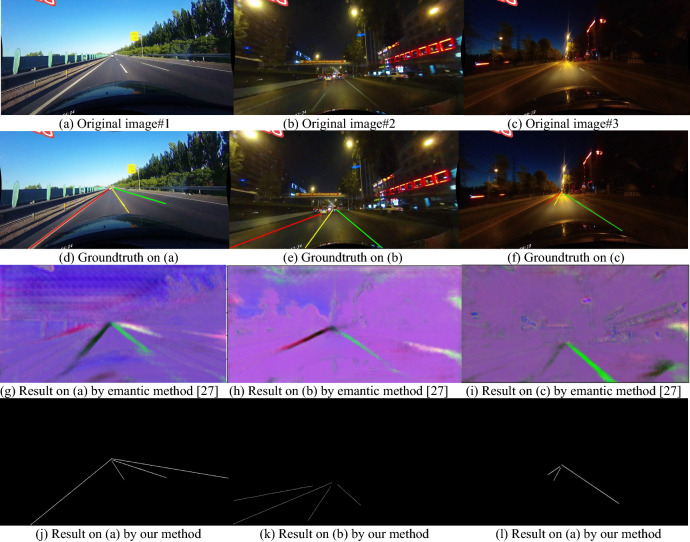
Detection results based on CuLane data set^44^.

To show the comparison results visually, we selected two groups of sample images from the two public dataset as following. We present three images from TuSimple data set^43^ and their detection results, and three images from CuLane data set^44^ and their lane line detection results. The testing results are described in Fig. 13 and Fig. 14 respectively.

In Fig. 13, the three original images are selected from the public dataset: TuSimple data set^43^, and the three images represent three different situations, they cannot be processed by a normal image segmentation algorithm because of complex of the images. By comparing to the current semantic method^27^, the new algorithm described above can give out the good lane line detection results for the straight lane lines. The details for the comparison are presented as the follows.

In Fig. 13a, it is a two lane road image, the left lane is a continuous yellow line, and the right lane is a white line but not a continuous line in which almost 70% length have no white color; comparing to the groundtruth in Fig. 13d, the semantic method (Deeplab V3 + network)^27^ creates 4 extra lane lines in addition to the two actual lanes as shown in Fig. 13g; and the new algorithm in this study can clearly detect the central lines of the two lanes as shown in Fig. 13j. The image in Fig. 13b has three lane lines, the middle lane line is short and not continuous, and the illumination in the image is uneven; the semantic method^27^ can detect the four lane lines (including the shoulder line on right), but also detects an extra short line on left, as shown in Fig. 13h; and the new algorithm can give out the three lane lines clearly, see Fig. 13k. In Fig. 13c, the image has three fuzzy and intermittent lane lines and multiple vehicles, and the illumination is uneven; expect for the lane lines, the semantic method also creates an extra lane line on left side of road as shown in Fig. 13h; and the studied algorithm in this paper can extract the three lane lines exactly, as shown in Fig. 13l.

In Fig. 14, one dusk image and two night original images are chosen from the public dataset: CuLane data set^44^, the three images are from different environments, as shown in their groundtruths, they are hard to deal with an existing lane line detecting algorithm based on image processing. The semantic method^27^ only gives out the rough results, and the lane lines cannot be clearly extracted, but the new algorithm can make the satisfactory results. The detailed explanation for the image segmentation and result comparison is analyzed in the following.

In Fig. 14a, it is a three lane dusk road image, the left lane line is a continuous yellow line, and the other lane lines are white lines but not continuous; comparing to the groundtruth in Fig. 14d, the semantic method^27^ creates 2–3 extra lane lines in addition to the three real lane lines as shown in Fig. 14g; and the new algorithm in this study can exactly detect the three central lane lines as shown in Fig. 14j. The image in Fig. 14b is a night image including strong lighters, it has three lane lines, the left lane line is vague, and the illumination in the image is uneven; the semantic method^27^ only detect the two lane lines of the three lane lines, but also extracts 1–2 extra short lines on right, as shown in Fig. 14k; and the new algorithm can give out the three lane lines exactly, see Fig. 14l. In Fig. 14c, the image is a night image with weak lighters, it has three lane lines, the two lane lines on left are very weak and short, the semantic method^27^ only identifies the long lane line on right, as shown in Fig. 14h; and the new algorithm can extract the three lane lines exactly, which are presented in Fig. 14l.

## Conclusion

The research content mainly includes five aspects:

1. Considering that the acquired lane line image at night, it does not necessarily have obvious lane lines, based on the length, width, interval distance of lane line (Table 1), possible vehicle speed (Table 2), road class and video acquisition frequency, a multiple video image fusion is made as the detecting image, in this way, the lane lines can be basically guaranteed showing up in each detecting image.
2. Since the influence of street lights at night on the traffic road is great, the detection region of lane lines cannot be a fixed for all the images, so a dynamic algorithm to determine the valid detection region is studied, which is an algorithm based on region merging.
3. Due to the low contrast and much noise of lane line images are acquired at night, an image smoothing and lane line enhancement algorithm is proposed based on Fragi and Hessian matrix based algorithm.
4. Because a lane line is 10–20 cm in width, the line is in white color, no matter what the worn line is, there are always some brighter points on the line. According to the feature, this paper suggests an algorithm to detect the feature points on the lane centerlines. After the image is inverted, based on the characteristics of the possible positions of the lane lines, the algorithm detects the valley edge points in four directions: if this point is judged to be a valley edge point, the maximum gradient magnitude value of its four directions is selected as the candidate point of the centerline feature point. By comparing Canny and the other edge detection operators, the new algorithm produces less noise, and most of the detected feature points are concentrated on or around the lane lines.

In the process of this study, hundreds of video images of lane lines on freeway at night are tested with 9 different algorithms, and the global thresholding, dynamic thresholding, different Canny edge detectors, Clustering analysis, fuzzy clustering analysis (FCM) and MST graph based algorithms are compared to the new method. The experimental and comparison results show that the new method (includes several algorithms) proposed in this paper can be applied for the automatic detection of the lane lines on the highway at night, and can achieve the good effect that other algorithms are difficult to obtain. Further research focuses on that the new method can automatically decide video image fusion rules for different length vehicles and different highway classes.

## Data Availability

All data generated or analyzed during this study are included in this published article.
